# Transpulmonary pressures in obese and non-obese COVID-19 ARDS

**DOI:** 10.1186/s13613-020-00745-w

**Published:** 2020-10-01

**Authors:** Mehdi Mezidi, Florence Daviet, Paul Chabert, Sami Hraiech, Laurent Bitker, Jean-Marie Forel, Hodane Yonis, Ines Gragueb, Francois Dhelft, Laurent Papazian, Jean-Christophe Richard, Christophe Guervilly

**Affiliations:** 1 Medical ICU, Croix-Rousse Hospital, Hospices Civils de Lyon, Lyon, France; 2 Lyon 1 University, Lyon, France; 3 Assistance Publique - Hôpitaux de Marseille, Hôpital Nord, Médecine Intensive Réanimation, Chemin Des Bourrely, 13015 Marseille, France; 4 Aix-Marseille Université, Faculté de médecine, Centre d’Etudes et de Recherches sur les Services de Santé et qualité de vie EA 3279, 13005 Marseille, France; 5 CREATIS, CNRS UMR5220, INSERM U1044, INSA, Lyon, France

## Abstract

**Background:**

Data on respiratory mechanics of COVID-19 ARDS patients are scarce. Respiratory mechanics and response to positive expiratory pressure (PEEP) may be different in obese and non-obese patients.

**Methods:**

We investigated esophageal pressure allowing determination of transpulmonary pressures (PL ) and elastances (EL) during a decremental PEEP trial from 20 to 6 cm H_2_O in a cohort of COVID-19 ARDS patients.

**Results:**

Fifteen patients were investigated, 8 obese and 7 non-obese patients. PEEP ≥ 16 cm H_2_O for obese patients and PEEP ≥10 cm H_2_O for non-obese patients were necessary to obtain positive expiratory P_*L*_. Change of PEEP did not alter significantly ΔP_*L*_ or elastances in obese patients. However, in non-obese patients lung EL  and ΔP_*L*_ increased significantly with PEEP increase. Chest wall EL was not affected by PEEP variations in both groups.

## Background

Obesity, which is usually associated with better outcome for acute respiratory distress syndrome (ARDS) patients, is considered as a risk factor of acquiring a severe form of SARS COV-2-associated ARDS for SARS-CoV-2 [1]. Impact of obesity on respiratory mechanics of SARS-CoV-2-associated ARDS has not been investigated.

We hypothesized that respiratory mechanics including esophageal pressure (*Pes*) measurements might be different in obese and non-obese patients.

Therefore, the first objective of this study was to investigate transpulmonary pressures (*P*_*L*_) in intubated SARS-CoV-2 patients according to their body mass index (BMI) during a decremental PEEP trial. Secondary objective was to assess lung and chest wall elastances (EL_*L*_and EL_*CW*,_ respectively).

## Methods

### Patients

We conducted a prospective observational study in two intensive care units both in tertiary university hospitals (Hôpital de la Croix-Rousse, Hospices Civils de Lyon and Hôpital Nord, Assistance Publique-Hôpitaux de Marseille).

Patients were included in the study from 15 March to 15 April 2020 if they fulfilled inclusion criteria: adult admitted into the ICU for SARS-CoV-2, intubated and mechanically ventilated with moderate-to-severe ARDS criteria, sedated and paralyzed for clinical purpose and monitored by *Pes* catheter. As part of routine clinical management, we performed a decremental PEEP trial from 20 to 6 cm H_2_O by 2 cm H_2_O-steps in each patient during volume-controlled ventilation while other parameters were kept constant.

### Esophageal pressure monitoring, transpulmonary pressures and elastances calculations

P*es* catheter (Nutrivent TM, Sidam, Mirandola, Italy, or C7680U (Marquat, Boissy-St-Leger, France) was in place. The correct placement of esophageal catheter was confirmed by presence of cardiac artifacts on the esophageal curve and by an occlusion test (expiratory hold on the ventilator) in passive conditions with gentle chest compression. The occlusion test was considered as positive when the correlation between ∆*P*_*es*_ and ∆ airway pressure (Paw) was 0.8–1.2. To avoid overestimation or underestimation of esophageal pressures, we inflated the esophageal balloon with the minimal filling volume among the recommended range for each catheter which was within the flat portion of the volume–pressure curve of the balloon [2, 3].

At each PEEP step, 2-s end-inspiratory occlusion pause allowed measurement of respiratory system (RS) and esophageal plateau pressure (Pplat and *P*_*es, insp*_, respectively), whereas 5-s end-expiratory occlusion pause allowed, respectively, measurement of RS and esophageal total PEEP (PEEP_*tot*_ and *P*_*es*,*exp*_ respectively). RS driving pressure (∆*P*_RS_) was calculated as Pplat minus PEEP_*tot*._ RS, chest wall and lung elastances (EL_*RS*_, EL_*CW*_, and EL_*L*_, respectively) and elastance ratio were computed according standard formula.

*P*_*L*_ absolute values were calculated as airway pressures minus esophageal pressures during inspiration (*P*_*L,insp*_ = *P*plat–*P*_*es,insp*_) and expiration (*P*_*L, exp*_ = PEEP_*tot*_–*P*_es,*exp*_), transpulmonary driving pressure (∆*P*_*L*_) = *P*_*L,insp*_–*P*_*L,exp*_. Elastance ratio derived *P*_*L*_ (*P*_*L,ER*_) was calculated as *P*plat x (EL_*L*_/EL_*RS*_).

### Statistics

Obesity was defined by a BMI ≥ 30. Results are reported as medians [interquartile range] or count (percentage) and compared between groups by Mann–Whitney U. Friedman test was used for repeated variables. *p* value < 0.05 was considered as significant.

### Results

**Fig. 1:**
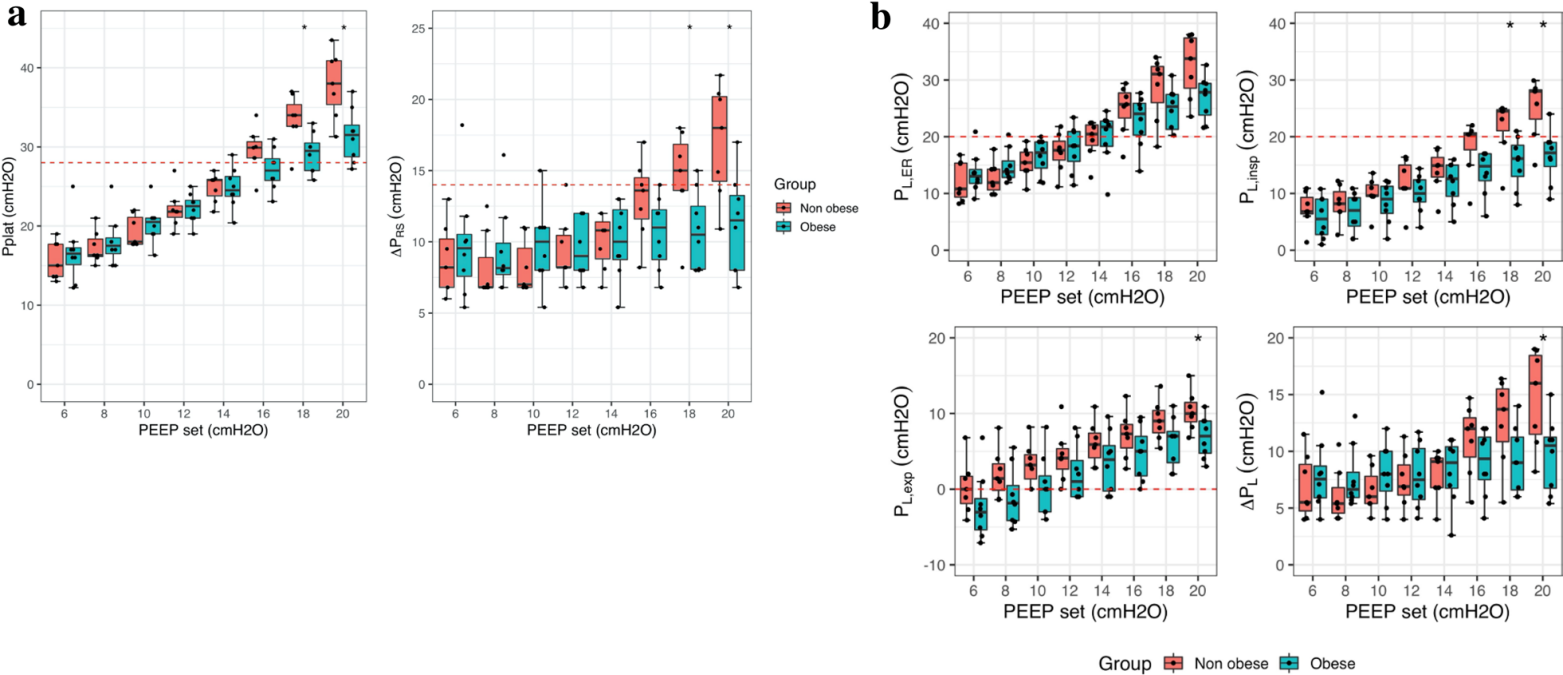
**a** Boxplots of respiratory system plateau pressures (Pplat) according to set PEEP and group. Black dots represent individual values. Red dashed line denotes 28 cm H_2_O. *p < 0.05 between obese and non-obese group for a given PEEP by Mann–Whitney U test. Boxplots of respiratory system driving pressures (∆P_RS_) according to set PEEP and group. Black dots represent individual values. Red dashed line denotes 14 cm H_2_O. *p < 0.05 between obese and non-obese group for a given PEEP by Mann–Whitney *U* test. **b** Boxplots of end-inspiratory elastance-ratio derived (*P*_*L, ER*_), end-inspiratory absolute method (*P*_*L, insp*_) and end-expiratory (*P*_*L, exp*_) transpulmonary pressures according to set PEEP and group. Black dots represent individual values. Red dashed lines denote 20 cm H_2_O for *P*_*L, ER*_and *P*_*L, insp*_ plots, and 0 cm H_2_O for *P*_*L, exp*_ plot. *p < 0.05 between obese and non-obese group for a given PEEP by Mann–Whitney *U* test

Fifteen patients were included in the study, 8 in the obese group (median BMI 34 [33–41]) and 7 in the non-obese group (mean BMI 26 [25–29]). Patient’s characteristics were comparable between groups except for age (66 [53–73] years for non-obese group vs. 44 [39–49] years for obese group, *p* = 0.04). Table 1 compares respiratory mechanics for each BMI group according to the PEEP levels. Figure 1a represents *P*plat and ∆*P*_RS_ for each BMI group according to the PEEP levels. Figure 1b represents transpulmonary pressures and ∆*P*_*L*_.

**Table 1:** Respiratory mechanics according to BMI group and PEEP level

VPariable	BMI group	PEEP (cm H_2_O)							
		6	8	10	12	14	16	18	20
*P*plat (cm H_2_O)	Non-obese^$^	15 [13.6–17.7]	16.3 [16.2–18.4]	18 [17.7–21.1]	21.8 [21.1–22.6]	25.8 [23.8–25.9]	29.9 [28.6–30.7]	*34 [32.6–35.4]*	*38 [35.4–40.9]*
	Obese^$^	16.5 [15.1–17.3]	17.5 [16.5–18.5]	20.5 [19–21]	22.5 [21–23.3]	24.5 [23.8–26.3]	27 [25.8–28.5]	29.5 [27–30.5]*	31.5 [28.8–32.8]*
Total PEEP (cm H_2_O)	Non-obese^$^	6.8 [6.8–6.9]	9 [8.4–9.5]	10.9 [10.9–11]	12.2 [12.2–13.3]	15 [15–15]	16.3 [16.3–16.7]	19 [19–19]	20.4 [20.4–21.4]
	Obese^$^	6.9 [6.7–6.9]	8.7 [8.3–8.9]	10.5 [10–11]	13 [12.2–13]	15 [14–15]	16.7 [16–17]	18.5 [18–19]	20 [20–20.6]
∆*P*_RS_ (cm H_2_O)	Non-obese^$^	8.2 [6.8–10.2]	6.8 [6.8–8.9]	7 [6.8–9.6]	8.2 [8.2–10.5]	10.8 [8.8–11.4]	13.6 [11.6–14.5]	*15 [13.6–16.9]*	*18 [14.3–20.2]*
	Obese	9.6 [7.6–10.5]	8.15 [7.7–9.9]	10 [8–11]	9 [8–12]	10 [8.8–12.3]	11 [8.8–12.3]	10.5 [8.1–12.5]*	11.5 [8–13.3]*
*P*_*es. insp*_ (cm H_2_O)	Non-obese^$^	8.6 [6.9–10.2]	9.4 [7.6–10.9]	10 [7.6–11.6]	10.9 [8.8–11.6]	10.9 [8.8–12.8]	10.9 [9.3–12.8]	12 [9.5–12.9]	13 [10.9–14.3]
	Obese^$^	11.5 [9.1–14.5]	11.5 [9.5–15.3]	11.5 [9.5–16.3]	12 [9.5–16]	12.5 [10.5–16.3]	12.5 [10.6–16.5]	13 [11.4–17.3]	13.5 [12.8–17.5]
*P*_*es. exp*_ (cm H_2_O)	Non-obese^$^	7.1 [5.2–8.9]	7.5 [5.9–8.9]	7.8 [6.4–10.2]	8.3 [7.1–10.2]	9.1 [7.1–10.9]	9.5 [7.6–11]	10 [8.1–11.6]	11 [8.8–12.2]
	Obese^$^	9.9 [8.1–12.3]	10.5 [8.2–13]	10.5 [8.5–13.3]	11 [8.7–14]	11 [8.7–15]	11.5 [9.3–14.5]	11.5 [10.3–15.3]	12.5 [11–16]
*P*_*L. insp*_ (cm H_2_O)	Non-obese^$^	6.8 [6.4–9.3]	8.2 [6.8–10.3]	9.6 [9.5–11.5]	10.9 [10.9–15]	14.9 [12.9–16.4]	20.4 [17.7–20.7]	*24.5 [21.1–24.8]*	*28 [23.1–28.3]*
	Obese^$^	5.5 [2.8–9]	7 [4.3–9.3]	9 [6.5–11.3]	10 [8.5–12.4]	12.6 [9.5–15]	14.8 [12.3–17]	16.2 [13–18.5]*	17.15 [14.8–19]*
*P*_*L, ER*_ (cm H_2_O)	Non-obese^$^	10.8 [9.4–15.4]	11.9 [10.6–14.3]	15.4 [14.1–17.3]	17.6 [15.9–19.2]	20.5 [18.5–22.1]	25.7 [23.3–27.7]	31.1 [26–32.4]	33.8 [28.6–37.4]
	Obese^$^	13 [11.8–14.2]	13.8 [12.8–15.7]	17.2 [14.5–19.1]	18.4 [15.7–20.9]	21.7 [18.3–22.8]	24 [20.3–25.9]	25.3 [21.3–27.4]	27.8 [23.8–29.4]
*P*_*L. exp*_ (cm H_2_O)	Non-obese^$^	0 [− 1.9 to 1.7]	1.4 [− 0.2 to 3.4]	3.2 [1.4–4.6]	4.1 [2.7–5.4]	5.9 [4.2–7.4]	7.3 [5.5–8.6]	9 [7.5–10.4]	*10 [8.9–11.5]*
	Obese^$^	− 3.1 [− 5.4 to − 1.3]	− 1.9 [− 4.2 to 0.5]	0 [− 3 to 1.8]	1 [− 1 to 3.8]	3.9 [− 0.3 to 5.8]	5 [1.8–7]	7 [3.5–7.6]	7 [4.8–9]*
∆*P*_*L*_	Non-obese^$^	5.5 [4.8–8.9]	5.4 [4.6–6.8]	6 [5.4–7.8]	6.9 [6.2–8.8]	9.1 [6.8–9.4]	12 [9.5–13]	13.7 [10.9–15.5]	*16 [11.5–18.5]*
	Obese	7.6 [5.9–8.7]	6.7 [5.9–8.2]	8 [6.5–10]	7.5 [5.8–10.3]	9 [6.8–10.4]	9.4 [7.5–11.3]	9 [6.6–11.3]	10.5 [6.8–11.3]*
EL_*RS*_ (cm H_2_O.L^−1^)	Non-obese^$^	20 [17.4–23.8]	18.3 [15.6–23.2]	18.3 [17–23.3]	24.1 [18.4–26.4]	24 [22.7–28.9]	33.3 [27.4–38.3]	39.3 [32–43]	47.1 [33.5–54.1]
	Obese	27.3 [18.5–38.1]	26.3 [18–38.3]	25.1 [17.6–53.7]	26.7 [18–49.1]	30.1 [18.6–48.5]	31.4 [19.9–53.4]	29.2 [23.3–45.9]	31.4 [22.9–45.9]
EL_*CW*_ (cm H_2_O.L^−1^)	Non-obese	4.6 [3.8–5.7]	5.8 [4.7–6.2]	3.2 [3.1–5.2]	5.2 [3.5–6.3]	5.2 [3.6–6.3]	5.2 [3.6–6.3]	3.8 [3.2–6.2]	6.2 [4.7–6.3]
	Obese	4.2 [4–5.7]	4.2 [3.5–7.1]	4.8 [2.7–7.8]	4.9 [2.7–8.2]	4.8 [4.2–8.4]	5.7 [2.7–8.2]	4.2 [3.5–6.7]	3.9 [2.7–6.7]
EL_*L*_(cm H_2_O.L^−1^)	Non-obese^$^	15.9 [11.6–20.7]	13.1 [10.9–17.8]	15.7 [13.7–19.6]	20.3 [13.8–22.2]	20.9 [17.7–24.6]	30.2 [22.5–33.8]	36.4 [25.7–39.8]	41.9 [27.2–48.7]
	Obese	23.3 [14.6–32.4]	22.3 [15.3–30.7]	22.6 [14.9–42]	23.2 [12.8–39.8]	26.4 [14.2–42.5]	28.9 [16.1–43.6]	25.2 [19.9–36.6]	28.9 [20.3–38.8]
EL ratio	Non-obese^$^	0.79 [0.67–0.87]	0.75 [0.66–0.80]	0.8 [0.79–0.84]	0.81 [0.74–0.84]	0.83 [0.74–0.85]	0.86 [0.8–0.9]	0.9 [0.79–0.92]	0.89 [0.81–0.9]
	Obese^$^	0.8 [0.77–0.85]	0.80 [0.78– 0.85]	0.84 [0.74–0.89]	0.82 [0.72–0.89]	0.85 [0.77–0.86]	0.87 [0.79–0.9]	0.85 [0.8–0.9]	0.88 [0.79–0.91]

Variables are reported as medians [interquartile range]

*BMI* body mass index, *PEEP* positive end-expiratory pressure, *Pplat* plateau pressure, *∆P*_*RS*_ respiratory system driving pressure, *P*_*es. insp*_ esophageal inspiratory pressure, *P*_*es. exp*_ esophageal expiratory pressure, *P*_*L. insp*_ inspiratory transpulmonary pressure, *P*_*L, ER*_ transpulmonary pressure calculated with the elastance ratio, EL ratio, *P*_*L. exp*_ expiratory transpulmonary pressure, *∆P*_*L*_ driving transpulmonary pressure, *EL*_*RS*_ respiratory system elastance, EL_*CW*_ chest wall elastance, *EL*_*L*_ lung elastance, *EL* ratio, ratio of lung elastance to elastance of the respiratory system.

*p < 0.05 between obese and non-obese group for a given PEEP by Mann–Whitney *U* test; ^$^*p* < 0.05 for PEEP effect by Friedman test. Computed for each group

PEEP ≥ 16 cm H_2_O for obese patients and PEEP ≥ 10 cm H_2_O for non-obese patients were necessary to obtain positive *P*_*L, exp*_ (Fig. 1b). At 16 cm H_2_O of PEEP, 71% of non-obese patients had *P*_*L*_, _*insp*_ ≥ 20 cm H_2_O and 0% of obese patients, whereas with *P*_*L, ER*_ was ≥ 20 cm H_2_O in, respectively, 86% of non-obese patients and 75% of obese patients. Change of PEEP did not alter significantly ∆*P*_*L*_or elastances in obese patients (Table 1). However, in non-obese patients EL_*RS*_ and EL_*L*_ increased significantly with PEEP increase. EL_*CW*_ was not affected by PEEP variations in both groups.

Differences between obese and non-obese groups were significant at 18–20 cm H_2_O of PEEP with higher *P*plat, ∆*P*_RS,_ *P*_*L,insp*_, ∆*P*_*L*_ in non-obese patients.

### Discussion

During decremental PEEP trial, we found differences in transpulmonary pressures and respiratory mechanics in COVID-19 ARDS patients according to the presence of obesity. First, *P*_*L,insp*_, ∆*P*_*L*_were higher in non-obese patients at high PEEP (≥ 18 cm H_2_O), as *P*plat and ∆*P*_RS_. Second, EL_*CW*_ and EL_*L*_ were not statistically different between groups. However, increase of PEEP was significantly associated with an increase of EL_*L*_ in non-obese patients. Third, high PEEP levels (i.e., 16 cm H_2_O) were associated with potential injurious *P*_*L,ER*_(≥ 20 cm H_2_O).

Preliminary studies with CT-scan have reported diffuse and bilateral pulmonary lesions during COVID-19 ARDS, which is usually associated with recruitability by PEEP in ARDS [4].

Recent studies using the same method to assess recruitability (recruitment-to-inflation ratio) reported conflicting data in those patients [5–7] with range from 17 to 56% of highly recruitable patients and possible less recruitable patients at a more advanced time point of the disease [5].

However, the lower driving pressures (∆*P*_*RS*_ and (∆*P*_*L*_) observed for obese patients cannot discriminate lung recruitment from less lung overdistension without appropriate evaluation (CT-scan or electrical impedance tomography for instance).

We did not check for airway flow limitation in all patients that could have led to overestimate lung and respiratory system elastances, in particular in obese patients.

Finally, we were not able to compare respiratory mechanics of this cohort with non COVID-19 ARDS patients.

In conclusion, assessment of respiratory mechanics of COVID-19 ARDS patients with transpulmonary pressure monitoring might be useful when targets of protective lung ventilation could not be reached. The characteristics of obesity on respiratory mechanics airway opening pressure, recruitability of COVID-19 ARDS patients need further investigations.

## Data Availability

The datasets analyzed during the current study are available from the corresponding author on reasonable request.

## Abbreviations

ARDS: Acute respiratory distress syndrome
BMI: Body mass index
∆*P*_L_: Transpulmonary driving pressure
∆*P*_RS_: Respiratory system driving pressure
EL_*CW*_: Chest wall elastance
EL_*L*_: Lung elastance
EL_*RS*_: Respiratory system elastance
ICU: Intensive care unit
PEEP: Positive end-expiratory pressure
*Pes*: Esophageal pressure
*P*_*L, ER*_: Inspiratory transpulmonary pressure (elastance ratio)
*P*_*L, exp*_: Expiratory transpulmonary pressure
*P*_*L, insp*_: Inspiratory transpulmonary pressure (absolute value)
*P*_*L*_: Transpulmonary pressure
*P*plat: Plateau pressure
SARS-CoV-2: Severe acute respiratory syndrome coronavirus
